# Will Passive Protection Save Congo Forests?

**DOI:** 10.1371/journal.pone.0128473

**Published:** 2015-06-24

**Authors:** Gillian L. Galford, Britaldo S. Soares-Filho, Laura J. Sonter, Nadine Laporte

**Affiliations:** 1 The Gund Institute for Ecological Economics, University of Vermont, 617 Main Street, Burlington, Vermont, 05405, United States of America; 2 Centro de Sensoriamento Remoto, Universidade Federal de Minas Gerais, Av. Antônio Carlos 6627, Belo Horizonte, 31270–901, Minas Gerais, Brazil; 3 The Woods Hole Research Center, 149 Woods Hole Road, Falmouth, Massachusetts, 02540, United States of America; DOE Pacific Northwest National Laboratory, UNITED STATES

## Abstract

Central Africa’s tropical forests are among the world’s largest carbon reserves. Historically, they have experienced low rates of deforestation. Pressures to clear land are increasing due to development of infrastructure and livelihoods, foreign investment in agriculture, and shifting land use management, particularly in the Democratic Republic of Congo (DRC). The DRC contains the greatest area of intact African forests. These store approximately 22 billion tons of carbon in aboveground live biomass, yet only 10% are protected. Can the status quo of passive protection — forest management that is low or nonexistent — ensure the preservation of this forest and its carbon? We have developed the SimCongo model to simulate changes in land cover and land use based on theorized policy scenarios from 2010 to 2050. Three scenarios were examined: the first (Historical Trends) assumes passive forest protection; the next (Conservation) posits active protection of forests and activation of the national REDD+ action plan, and the last (Agricultural Development) assumes increased agricultural activities in forested land with concomitant increased deforestation. SimCongo is a cellular automata model based on Bayesian statistical methods tailored for the DRC, built with the Dinamica-EGO platform. The model is parameterized and validated with deforestation observations from the past and runs the scenarios from 2010 through 2050 with a yearly time step. We estimate the Historical Trends trajectory will result in average emissions of 139 million t CO_2_ year^-1^ by the 2040s, a 15% increase over current emissions. The Conservation scenario would result in 58% less clearing than Historical Trends and would conserve carbon-dense forest and woodland savanna areas. The Agricultural Development scenario leads to emissions of 212 million t CO_2_ year^-1^ by the 2040s. These scenarios are heuristic examples of policy’s influence on forest conservation and carbon storage. Our results suggest that 1) passive protection of the DRC’s forest and woodland savanna is insufficient to reduce deforestation; and 2): enactment of a REDD+ plan or similar conservation measure is needed to actively protect Congo forests, their unique ecology, and their important role in the global carbon cycle.

## Introduction

Changes in land use, such as deforestation and forest degradation, are a major source of worldwide carbon dioxide emissions, second only to the burning of fossil fuels [1]. Tropical ecosystems are the primary source of emissions from land use change and the greatest uncertainty in emissions stems from African tropical forests [2]. Carbon emissions in the tropics come primarily from three hotspots: first, industrial-scale agriculture and cattle ranching in the Amazon; next, industrial palm oil plantations in SE Asia; and finally, household slash-and-burn agriculture in Africa [3]. Deforestation in Africa accounts for 19% of greenhouse gas emissions from land use change [4] but has gained little attention to date, although African forests as the second largest aboveground carbon reservoir in the world [5]. African forests experience a net loss of 3.4 million ha year^-1^, a rate that has remained stable over the last decade, but is likely to accelerate rapidly in the near future as demands for timber products and agricultural land increase [6]. The Democratic Republic of Congo (DRC) is the frontier of deforestation in Africa [7] where 22 billion metric tons of carbon are stored in the aboveground live biomass of its forests and woodland savannas [5]. Rapid deforestation here will not only lead to local losses of ecosystem services and goods, it may also result in large carbon emissions.

In the DRC, patterns of deforestation and its direct and indirect causes were not well understood in academic or policy literature until the 2000s [7–9]. Widespread mining and commercial logging are now known to be direct causes of forest loss. The spatial correlation between mining and commercial logging is low, as their footprints are limited to areas much smaller than the extent of deforestation observed by Potapov et al. [10]. Small-scale, direct factors for deforestation include extensive smallholder farming and harvest of trees for charcoal and fuel. Smallholder clearings often nucleate around commercial logging and mining as new road infrastructure provides fresh access to tracts of forest. Demands on forests from smallholder farms are likely to increase in coming decades due to a growing population whose food supply is increasingly insecure. The extent of hunger in the DRC has gone from “alarming” to “extremely alarming” over the last two decades [11] as population doubled. By 2025, it is expected to increase ~150% from its current figure of 74 million [12]. An increasingly hungry, growing population may turn to clear cutting forests and savanna woodlands to produce more food (extensification). The pressure to reduce rotation times for fields will lead to declines in soil fertility. In the DRC, dependence on subsistence farming, combined with low market access for the rural poor, encourages extensification and unsustainable use of forest resources.

Indirect factors influencing deforestation are less clear. Development efforts may influence the way land and forest resources are used (e.g., lack of capital to invest in improved agricultural techniques or alternative energy sources). Technological factors may limit efficiency in resource use; for example, access to improved hybrid seeds, which boost food production, may be lacking. Cultural factors can play a role, such as little or no awareness of the impacts of degradation on livelihoods. Lastly, political and institutional factors can play an indirect role in deforestation (e.g., lack of monitoring or enforcement.) [9].

In addition to these endogenous pressures, African nations are experiencing a range of worldwide demands for resources, and their own national desires for capital. Recent literature has publicized so-called African “land grabs,” where foreign corporations and investors are funding large-scale, industrialized agricultural operations that include conversion from forests or savanna woodlands [6, 13, 14]. Between 2007 and 2010 in the DRC alone, over 11,000 ha were part of such land deals, [13] although clearing has yet to occur due to issues involving permits and funding. Additional global land pressures include the rapid increases in demand for biofuels that could lead to substantial sub-Saharan deforestation in the near future [15]. Hence, African deforestation rates are expected to increase in the coming years through global pressures that include increased demands for food, timber and biofuel production.

The DRC has moved toward political stability in recent years and must now focus on managing the huge economic potential of its natural resources. Historically, forests had “passive protection,” a sort of *de facto* conservation with low deforestation rates. This was due to chronic political instability and conflict, inadequate transportation and agricultural infrastructure, and poor governance. As a result, many areas designated “protected” existed, but had no governmental monitoring or enforcement of deforestation policies and thus were protected only by their remote location. Today, policy interventions could influence future forest development; they may range from over-exploitation to sustainable use.

New policies are underway to conserve forests, support sustainable economic development, and alleviate poverty simultaneously. One such effort is the DRC’s National Framework Strategy for the United Nations Reduced Emissions from Deforestation and Degradation Programme (REDD+) [16]. This framework pursues policies to increase the sustainable use of forests; it includes expansion of protected areas, zoning for land uses, reducing illegal logging, managing forests for biodiversity, increasing development in rural communities to reduce deforestation there, and limiting urban and industrial impacts on forests. For example, the DRC intends to increase protected areas from 10% of the country to 15% [8], although the time frame and criteria for protected areas are not yet well-determined [16].

In this study we calibrated a spatially explicit land-use change model based on 20 years of remote sensing observations, and simulated future land-use change under three scenarios. We examined three questions: (1) Will continued passive protection of forests aid in conservation? (2) Can the implementation of conservation policies (e.g. REDD+) significantly reduce carbon emissions? (3) To what extent would increased deforestation rates impact forest cover and carbon storage?

## Methods

### Study Area

**Fig 1:**
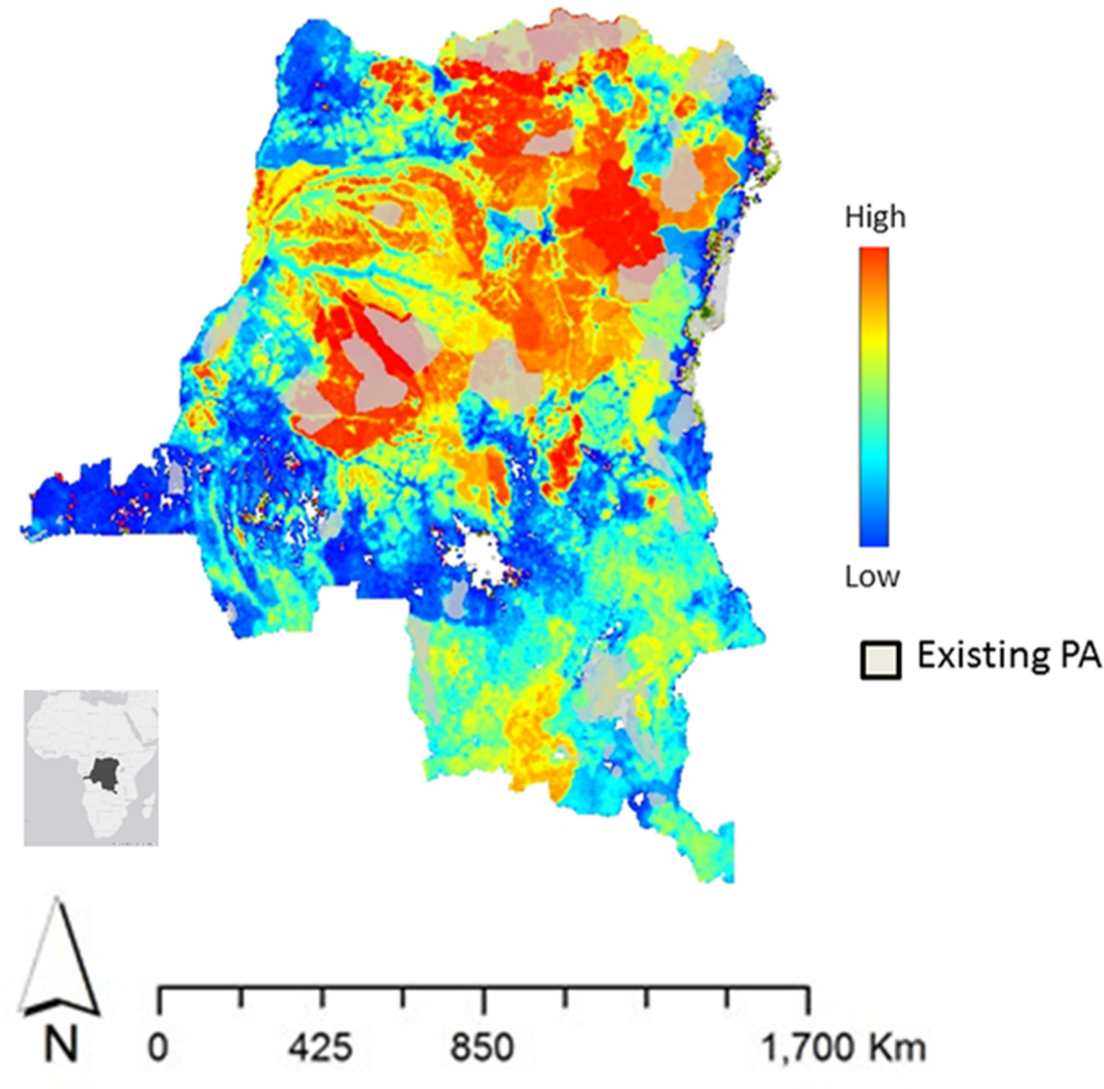
Favorability for protected areas estimated by rural population density and carbon biomass.

The DRC covers 2.3 million km^2^, of which 1.1 million km^2^ are forest and 0.6 million km^2^ are woodland savanna (Fig 1). The central and northern regions experience humid tropical climate, which transitions to slightly cooler and drier weather in the southern highlands. The population is approximately 74 million and the average rural population density is 0.2 people km^-2^ [17]. Few major roads are paved and many are in disrepair. Major waterways, such as the Congo River, serve as an additional transportation pathway to move both goods and people.

### Land-use change data

We combined several data sources to create a comprehensive map that quantifies the extent of land-use change observed in the DRC between 1990 and 2010. Categories included forest types (forest and woodland savanna), cleared lands, croplands, and plantations. We studied forests and woodland savannas individually, since they differed in deforestation rates and in the processes driving deforestation. However, the DRC has combined both forest types into a single class that is defined as any area with a crown cover of at least 30% [10].

Maps of forest types and cleared land were provided by Potapov et al. [10] for the three observation periods:1990–2000, 2000–2005, 2005–2010 (Table 1). This data set is the most thorough evaluation of land-use change in the DRC to date. To establish the extent of agriculture, we used a 2008 map of croplands provided by the geo-wiki.org group at International Institute for Applied Systems Analysis, who used the best classification agreement from five commonly used global data sets [18]. This land-use class was used to evaluate the influence of agriculture on deforestation rates. Lastly, plantations (including forest crops and oil palm) were mapped by the Woods Hole Research Center in 2010, using Google Earth imagery and following the methodology used to detect deforestation and roads by Laporte et al. [7].

**Table 1:** Annual rates of deforestation in primary forest and primary savanna reported by Potapov et al. [10].

Clearing (km^2^ year^-1^)	1990–2000	2000–2005	2005–2010
Forests	2,158	698	1,372
Woodlands		215	355

Deforestation (conversion of forest types to cleared land) was mapped by comparing the Potapov et al. maps [10] over the three time periods (Table 1). We found some minor errors in this data set, such as areas mapped as deforested in the 1990–2000 time period were subsequently classed as woodlands or primary forest. To improve consistency, we delineated the extent of each forest type in 2010 using MODIS Vegetation Continuous Fields definitions (VCF) for forest (>60% tree cover) and woodland savannas (30–60% tree cover) [19]. These were also used by Potapov et al. [10]. Woodland savannas, however, were not mapped prior to 2000. Therefore, to map deforestation of woodland savannas during the 1990–2000 decade, we took the ratios of forest to woodland savanna deforestation during the 2000–2010 period, and applied these ratios to deforested areas in the earlier decade. This assumed that deforestation was proportional for forest and woodland savannas. While this may not be precise, it did provide a good first-order estimate, since deforestation rates were relatively low during this early period. Finally, where cleared land and cropland overlapped in our dataset, we assigned the pixel first to the deforestation rate, and then to establishing the correlation between croplands and deforestation (see Section IV).

### The SimCongo Model

We developed the SimCongo model to analyze land use change in the DRC. The model was built on the freely available Dinamica-EGO platform [20], which has been widely used to model deforestation (e.g., [21, 22]). The SimCongo model and its parameters are available online: http://go.whrc.org/simcongo. Instructions for its use may be found through the Dinamica-EGO webpage and wiki (http://csr.ufmg.br/dinamica/). The model operates within two spatial structures: (1) a spatially explicit model allocating land use change at the pixel scale (Section IV), and (2) a scenario model projecting future rates of land use change for the DRC (Section V).

#### Spatially explicit model

We calibrated the spatially explicit model for the 1990–2010 time period using the deforestation measurements of Potapov et al. [10] (Table 1). We converted these gross deforestation rates to annual rates for both forest and woodland savannas, using the transition matrix function in Dinamica-EGO (Fig. A in S1 File). The spatial probability of deforestation was calibrated using the Bayesian Weights of Evidence (WofE) method [23–26] (S1 File).

WofE coefficients were calculated for a set of spatially distributed explanatory variables to determine their effect on deforestation. Fourteen variables were significant, so these were used in our model calibration (Table 2; S1 File). We found deforestation probability declined with increasing distance to roads, rivers, towns and villages, and with increasing slope. The probability of deforestation increased gradually with increasing distance to croplands until it began to drop at distances greater than ~1 km from croplands. Distance to railroads had a linear, positive relationship to deforestation probability in savanna woodlands and a “U” shape in forest regions. Deforestation probability had an inverted “U- shaped" relationship with biomass in savanna woodlands, where deforestation was greatest in mid-ranges, and a declining relationship with biomass in forests. Soil types [28] and vegetation sub-types are categorical variables; each category had an independent correlation with deforestation probability. None of these variables were spatially autocorrelated (S1 File). It is interesting to note that some variables we tested were not correlated with deforestation and therefore not used in the model. For example, the spatial distribution of small secondary, tertiary, and logging roads, or mining and logging concessions, did not increase the probability of deforestation. Similarly, agricultural suitability (represented by agro-ecological zones [35] and soil fertility [36]) did not influence deforestation.

**Table 2:** Spatial variables driving the model that have strong correlation to deforestation and low spatial autocorrelation.

Variable in SimCongo	Description of input
Woodland & forest cover	MODIS Vegetation Continuous Fields [19]
Vegetation types	FAO AfriCover [27]
Soils	FAO Harmonized World Soils Database [28]
Distance to railways	CARPE Railroad [29]
Distance to towns	CARPE Town populations [30]
Elevation	SRTM Elevation [31]
Distance to major rivers	CARPE River types [32]
Distance to roads	WHRC and WRI All road types, many digitized by hand [7, 33]
Distance to villages	CARPE Village populations [30]
Distance to deforestation	This paper
Population density	AfriPop [17]
Slope percent	SRTM Elevation [31]
Wetlands	Global Land Cover [34]
Cropland areas	Geo-wiki.org [18]

The WofE coefficients and the spatial distribution of calibration variables were combined to produce a deforestation probability map for both forest types. We validated these maps by simulating deforestation from 1990 to 2010, using historic deforestation rates (S1 File). The model allocated deforestation at the pixel scale according to the deforestation probability map, and a seeding mechanism allowed deforestation expansion from previously cleared land and establishment in new patches (S1 File). We compared the simulated 2010 land use map with the observed 2010 land use map, using fuzzy logic and an exponential decay function at multiple increasing windows [37]. We found a fit of 78% within a 3.5 km search radius; this improved to 90% when widened to 5.5 km (Fig. D in S1 File).

#### Scenario models

We developed three land-use change scenarios to simulate future possible deforestation trajectories. These scenarios were entitled Historical Trends, Conservation, and Agro-industrial Development. Each had a unique deforestation rate, and differed in its spatial allocation of land use change (as described in the following sub-sections). Future land use change was simulated for each scenario from 2010 to 2050, with annual iterations.

#### Historical Trends Scenario

The Historical Trends scenario (HT) represents a future of continued passive protection. Therefore, we used observed historical deforestation trends (1990–2010; Table 3) to project future deforestation (ha year^-1^) to 2050. A linear regression model (LabFit Pro freeware [38]) projected deforestation of forest and woodland savanna, where ***t*** is the time in years from 2010:

$$HT_{Forest}=\left(117\;\times t\right)+53.2\;\;\;\;\;\left(R^{2}=0.99\right)$$ (1)

$$HT_{Savanna}=\left(27.9\;\times t\right)+187$$ (2)

**Table 3:** Modeled scenarios of future land cover change with average rates of forest and savanna clearing, including secondary forest conversions.

Scenario	Deforestation rates	Average Rates (km^2^ year^-1^)
Historical Trends (HT)	Trend derived from the long-term, observed average rates (1990–2010)	2, 676
Agro-industrial Development (AD)	Interpolation based on the highest observed rates (2005–2010); Accounts for additional clearing for oil palm in the forest and industrial agriculture in woodland savannas	3,430
Conservation (CON)	Projection from long-term, observed average rates (1990–2000) are modified by decreasing rural population density as a surrogate metric for an effective REDD+ policy implementation for more “sedentary” lifestyles, agricultural intensification, and increased crop productivity	2,158

These regression models were selected through use of LabFit Pro [38], which ranks up to 300 possible curve fits. Eq 1 was selected because of its high R^2^ value. For Eq 2, only two data points were available [10] so an R^2^ value of one would misrepresent the strength of the model; these observations are the only spatial estimates of deforestation in the DRC’s woodland savannas.

Projected annual deforestation rates will increase linearly between 2010 and 2050 (Table 2) in response to all significant constant pressures, such as population growth that creates demands for food, firewood and charcoal. However, deforestation typically follows an inverted U-shaped curve [39], where national rates eventually peak and decline. This has been accurate in Africa roughly three-fourths of the time [40], where deviations can be caused by rural poverty traps, war, and expanding markets [40]. Given that all of these factors have been present during the DRC’s recent history, and since historic deforestation rates are low relative to the extent of remaining forest, it is unlikely rates will peak before 2050 in a scenario of continued passive protection.

### Conservation Scenario

The Conservation scenario (CON) represents a future of increased preservation. It was designed to capture the effects of multiple conservation policies recently proposed in the DRC [16]. Implementation of these policies would include activities such as expanding protected area networks [41], establishing (and enforcing) new land use zones [16], and land sparing through intensification and consolidation of subsidence farming [16]. Each policy aims to be easily capable of implementation and would achieve significant reductions in carbon emissions; however, the mechanisms for such implementation are not elucidated in the DRC’s policy statement [8, 16]. Therefore, we designed the CON scenario to capture the proposed effects of these multiple policies on national deforestation rates and spatial distribution of deforestation. This was done in three ways: first, by expanding conservation areas; next, by diverting deforestation to marginal agricultural land, and finally, by modifying subsistence farming practices.

As shown in the REDD+ plan, the DRC intends to expand protected areas so they occupy 15% of the country [16]. To capture this effect, we updated the DRC’s protected area map used by the spatially explicit model. Our criteria for new protected areas were low population density and high biomass; these align with DRC objectives to mitigate carbon emissions [16]. We ran 10 simulations of new protected areas and combined these results to create a conservation probability map. We mandated a minimum protected area of 21,500 km^2^ in order to constrain the number of new protected areas, and we set the new total protected area to 15% of the country.

Another DRC proposal aims to shift deforestation for agricultural use from dense forests to marginal zones [16]. The objective of these changes is to reduce carbon emissions per unit of deforestation; however, these “marginal zones” are not yet defined. If woodland savannas are considered marginal (since they have low aboveground carbon content relative to forests [5]), diverting deforestation to this forest type poses other implications for biodiversity and ecosystem services [42–43]. In this study, we defined marginal zones as those lands already cleared, so as to avoid negative tradeoffs. We captured the effects of zoning in our model by decreasing the relative probability of deforestation in forests and woodland savannas with high biomass.

Finally, the DRC has proposed to increase the productivity of subsistence farmers by implementing “sedentary” lifestyles through large-scale farming programs [16]. This policy aims to have 50% of subsistence farms in large-scale agricultural projects by 2030. It also aims to intensify production of at least 75% of these farms during this time. These changes are intended to reduce deforestation pressures by increasing production on lands already cleared, and by slowing population growth in outlying areas [16]. To capture these effects in our model, we used rural population density decline as a proxy for declining deforestation pressure. We related population density [17] and deforestation using a linear regression model in LabFit Pro, and decreased population density over time, using the following decay function (Fig. C in S1 File):

$$Population\;modifier=\left(0.1084\;x\;pop.\;density\right)+0.2133\;\;\;\;\;\left(R^{2}=0.65\right)$$ (3)

We then used rural population density (people per km^2^) [17] as a constraint to build this relationship [38]. We forced it to decrease to half its 2010 value by 2050 to support the farming goals mentioned previously [16]. Therefore, annual deforestation rates for the CON scenario (COND) were reduced from the HT rates (Eqs. 2–3) by the population modifier:

$$COND_{Forest}=\;HT_{Forest}÷population\;modifier$$ (4)

$$COND_{Savanna}=\;HT_{Savanna}÷population\;modifier$$ (5)

Therefore, the CON scenario assumes all other forces driving deforestation that were captured in the HT scenario remain constant in the CON scenario.

### Agro-industrial Development Scenario

The Agro-industrial Development scenario (AD) represents a future of increased deforestation rates due to commercial agricultural expansion, including both large scale oil palm plantations and industrially farmed crops. Growing global demand for oil palm for cooking and biofuels has recently increased the DRC’s annual production from 175,000 tons in the 1990s to 215,000 tons in 2010 [44]. Increasing demand for agricultural products is likely to continue, and given the recent investments in Africa’s (and the DRC’s) agricultural sector [6, 13, 14, 15], increases in production will likely add to pressures to expand cultivated areas through deforestation. This is especially true if the conservation initiatives (utilized in the CON scenario) are not implemented or enforced. Therefore, to incorporate these agro-industrial effects in our model, (1) we projected increased future deforestation and agricultural expansion rates, and (2) created a new spatial probability map to allocate deforestation for agriculture and situate it in the landscape.

Future annual deforestation rates (AD) were derived from observed historical rates. The 2005–2010 time period was used to capture the recent increased rates due to growing global demand for agricultural products as well as investments in the country’s agricultural sector (Table 1), and these rates were extrapolated using LabFit Pro as a function of time (**t**):

$$AD_{Forest}=\left[\left(135\;\times t\right)+561\right]\;\times f$$ (6)

$$AD_{Savanna}=\left[\left(25.3\;\times t\right)+239\right]\;\times f$$ (7)

Future annual agricultural expansion rates (AD_Ag_) were derived using a linear regression in LabFit Pro of annual, national scale increases in croplands (including oil palm, cereals, grains, fiber, pulses, and root vegetables) from 2000 to 2010 as reported by the FAO [37]:

$$AD_{Ag}=\left(32.3\;\times t\right)+1630$$ (8)

Agricultural expansion can also cause feedback (***f***) to Eqs 6 and 7 that modifies future deforestation rates. For example, large scale forest clearing has been observed to attract settlers who increase the local population density. This elevates local demands for agricultural lands, homesteads and forest products, and thus leads to additional deforestation [6]. We projected a constant growth rate in population density based on the latest reported rate (2.579%) [12]. To capture this effect in our model, we modified projected deforestation rates ***i*** using the feedback ***f*** (Eq 9), where ***x***_***1***_ is population density, ***x***_***2***_ is distance to croplands and ***x***_***3***_ is distance to roads:

$$\text{f}=0.7+\left(0.09\times {\text{x}}_{1}\right)-\left(6.1÷{\text{x}}_{2}\right)-\left(0.6\;\times 10^{-4}\times {\text{x}}_{3}\right)\;\;\;\;\left({\text{R}}^{2}=0.96\right)$$ (9)

This equation suggests that deforestation rates in the DRC increase with increasing population density, decrease with increasing distance to croplands, and decrease with increasing distance to roads. The R^2^ value (0.96) indicates that these three factors explain most of the variability in deforestation. These variables were tested as described by [20] and were found not to be spatially autocorrelated [22].

Finally, we created a spatial probability map of agricultural expansion in the DRC for use by the spatially explicit model. The current extent of cropland was determined from the data set in Fritz et al. [18] and used to parameterize the model to find suitable areas for expansion. Using the WofE method, we determined explanatory variables of historic agricultural expansion. We found that this expansion was correlated with areas of continuous slope), steepness [31], soil types [28, 38], existing croplands (all types) [18], distance to roads [7, 33], and distance to rivers [32]. To simulate agricultural expansion, SimCongo’s transition functions [22] were set to reflect plantation patch size (typically 1,000 ha; S1 File). Since cropland maps were available for only one time period, validation of this probability map was not possible; however, our correlated variables agreed with those previously published in literature, as cited above for each variable.

### Future carbon emissions

We quantified carbon emissions from projected future land use change (2010–2050) using the aboveground biomass map of Baccini et al. [5]. We assumed the carbon content of aboveground biomass is 50%, and that 85% of aboveground carbon is lost to the atmosphere during clearing operations [45]. In the CON scenario we reduced the quantity of carbon lost to 60% to capture the proposed effects of improved harvest practices, including chop-and-mulch instead of slash-and-burn, practices now being tested in the Amazon [46]. This is a conservative estimate of carbon losses from decreased harvest loss; the actual savings could be greater. The aboveground biomass map has an accuracy of 19 Mg C ha^-1^ for Africa [5]. We modified the biomass estimates up- and down-wards by the error range to estimate upper and lower bounds are carbon emissions.

## Results

### Protected Areas

**Fig 2:**
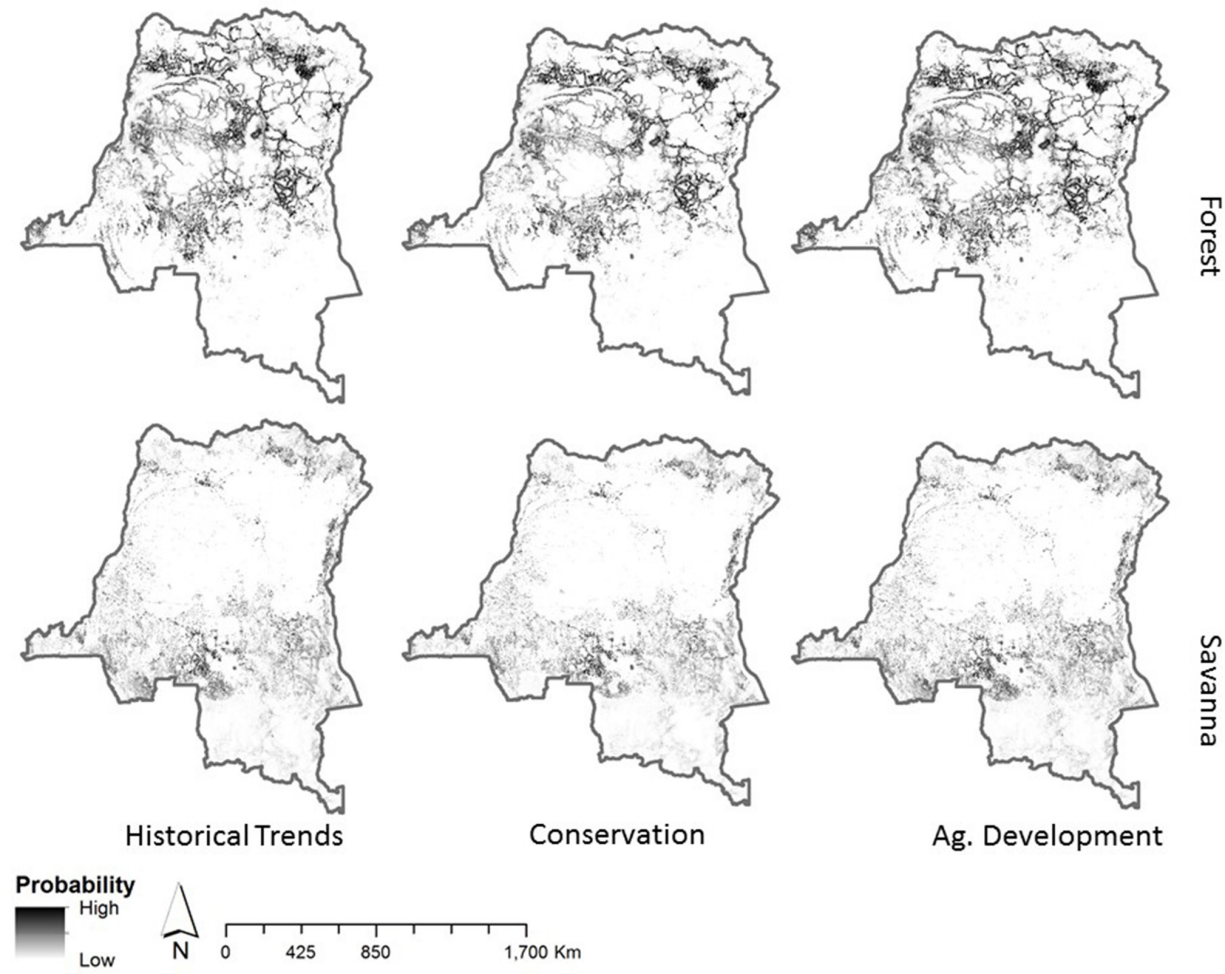
Probabilities of deforestation in forest and savanna by scenario.

The probability map derived for new protected areas (Fig 2) using the CON scenario criteria demonstrates that there is potential to increase conservation efforts. Specific opportunities to increase protected areas exist in the central forest near Salonga National Park, Sankuru Nature Reserve and Oshwe Game Reserve, and in the northern forest near Maiko National Park. This map also shows there is potential to increase protected areas in woodland savanna within the southern highlands.

### Land clearing

The probability of deforestation differs by scenario and is updated annually. To illustrate these differences we present deforestation probabilities for savanna and forest in Fig 1. Large tracts of forest have low probability of deforestation in the CON scenario. The probability of deforestation in remote areas spreads in the AD scenario. In all cases, the probability of deforestation clearly shows the strong dependence on infrastructure, existing deforestation and developed areas and river access.

**Fig 3:**
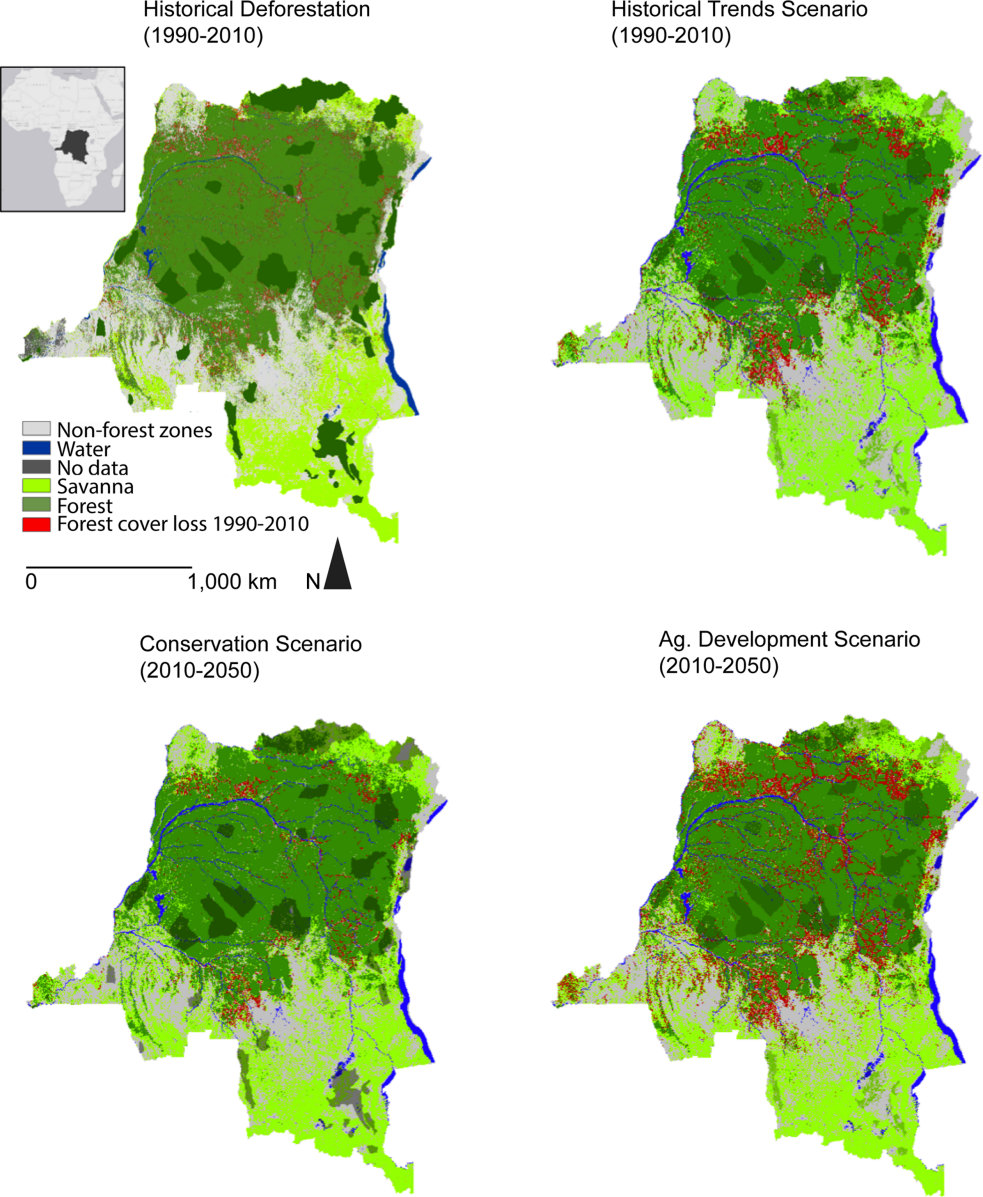
Deforestation extent under the Historical Trends, Conservation (including development of new protected areas based on population density and carbon in aboveground biomass), and Agricultural Development scenarios (including deforestation for croplands and oil palm).

Across all scenarios, deforestation is simulated to occur in 25–30% of the DRC by 2050, producing a total deforested area of 0.5 to 0.7 million km^2^ (Table 4). In all scenarios, deforestation is greatest near areas with high population densities, roads, rivers and areas of median biomass. Under HT (Fig 3), annual deforestation rates are estimated to reach 2,065 km^2^ year^-1^ by the final decade of the study. By comparing the CON (Fig 3) and HT scenarios, we find that by shifting population densities to favor lower density in rural areas, 58% less clearing is achieved. In the CON scenario, annual deforestation rates average 800 km^2^ year^-1^ by the 2040s. In the AD scenario, vast areas in close proximity to roads are used for deforestation once there are no limits to forest access (AD, Fig 3). In this scenario, annual average deforestation rates reached 5,393 km^2^ year^-1^ in the 2040s. In all scenarios, deforestation is largely driven by proximity to rivers, roads and previously deforested lands.

**Table 4:** Cumulative deforestation from forest and woodland savannas by 2050 and associated carbon emissions (2010 to 2050).

	Losses by 2050		
Scenario	Forest (km^2^)	Woodlands (km^2^)	Carbon (billion t CO_2_-e)
HT	98,280	35,600	3.8
CON	41,650	13,219	1.5
AD	155,480	45,635	6.1

### Carbon Emissions

**Fig 4:**
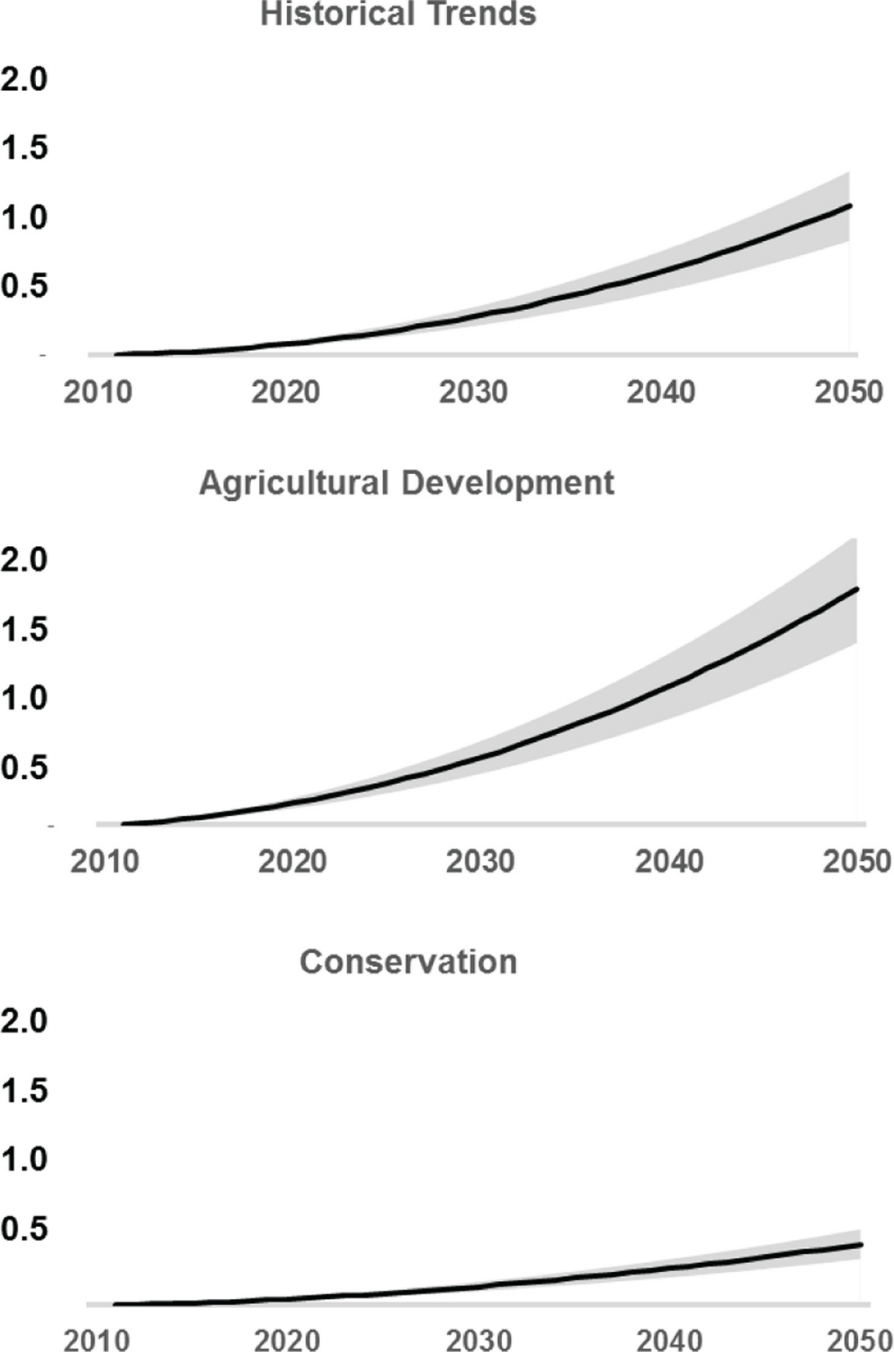
Carbon losses with uncertainties for each scenario.

The HT scenario illustrates a future where deforestation and associated carbon emissions remain at 37.8 million t C year^-1^ (139 million t CO_2_ year^-1^) through the 2040s. The cumulative emissions from deforestation would reach 3.8 billion tons CO_2_-e by 2050 (range: 2.9 to 4.7 billion tons CO_2_-e) (Fig 4). It is important to note that the estimates from the HT scenario are not meant to be used as a baseline looking forward, since land use trajectories can change at any point. Rather, it is an estimated representation based on present patterns. Currently, the DRC is creating its baseline reference [8].

REDD+ efforts to reduce carbon emissions (CON scenario) may further reduce that figure to 13.3 million t C year^-1^ (49 million t CO_2_ year^-1^), resulting in net carbon storage of 56.4 million t C in aboveground biomass, compared to the HT scenario (Table 4). The cumulative losses through 2050 would be 1.5 billion tons CO_2_-e (range: 1.1 to 1.8 billion tons CO_2_-e) (Fig 4).

**Fig 5:**
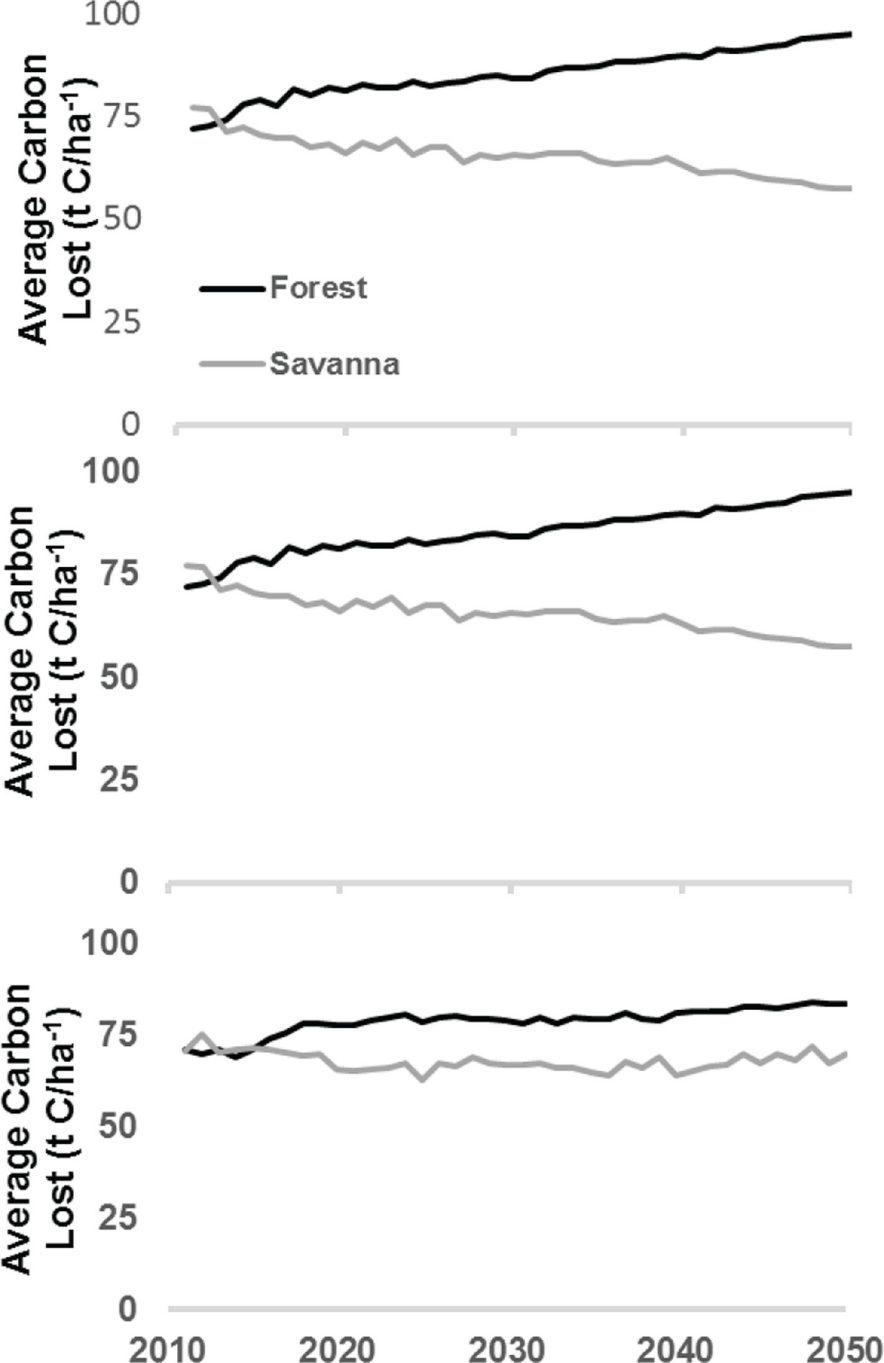
Average carbon loss over time shows trends towards increasing or decreasing carbon stocks depending on the location and scenario assumptions.

In the AD scenario, carbon emissions almost triple: the presence of increased industrial agriculture and oil palm development reaches 57.8 million t C year^-1^ or 212 million t CO_2_ year^-1^ by the 2040s. Cumulative losses through 2050 would result in an emission of 6.1 billion tons CO2-e (range: 4.4 to 7.1 billion tons CO_2_-e) (Fig 4). New deforestation moves into progressively higher biomass areas as more forest is cleared (Fig 5). However, preference is first given to lower biomass areas near roads and villages.

## Discussion

### Protected Areas

The probability map for future protected areas is based mainly on carbon density. Incorporating additional criteria, such as data on biodiversity, ecosystem services, and alternative uses would enhance this product for planning purposes.

In the DRC and, in fact, in all countries, it is essential that protected areas not only exist, but that conservation laws be observed and enforced if preservation is to succeed. The DRC’s capacity for monitoring deforestation and forest conservation will increase through its REDD+ readiness program. At the national level, a land use monitoring system for rapid assessment of forest change is under development using the Brazilian deforestation mapping system; land use monitoring, when paired with land based enforcement and better governance, is an efficient and effective way to administer conservation laws. Funding through REDD+ programs or green development funds may advance these conservation efforts, especially if sustainable rural development programs are a focus.

### Land Clearing

The CON scenario demonstrates the impacts and benefits of reduced rural deforestation. Alleviating rural pressures on forests would require large investments in agricultural development and alternative livelihoods for the rural poor. These would benefit both rural development and carbon storage. While the success of these investments is uncertain, it is important to consider development policies that also mitigate unsustainable pressures on natural resources. Today, rural areas in Malawi, Kenya, Nigeria and Mali are targeted for agricultural subsidies that reduce extensification and the need for woodland exploitation; similar programs can be developed in the Congo as part of REDD+. However, it would take large capital investments for the DRC to realize this type of development.

### Carbon emissions

We assess potential means of storing carbon by comparing the results of the three scenarios. Primary forest represents the largest initial land cover and greatest biomass, so protection of these areas has the most significant direct impact on emission reduction. The CON scenario illustrates that the proposed policy mechanisms (e.g., abating pressure to clear forests through diversified rural livelihoods) can result in lower carbon emissions. Incentives such as protected corridors or buffers around roads could alleviate pressures on forests and minimize emissions [34]. If deforestation is unavoidable, and lowering carbon emissions is a priority, low biomass forests should be chosen, as outlined in the DRC’s REDD+ strategy [12].

In addition to this study, a modest number of other analyses on potential carbon emissions from the DRC have been made. The DRC recently released its first estimates of future carbon emissions as guidance for REDD+ policies on reference emission levels [12]. They use a bookkeeping approach that assumes all forested areas have an aboveground biomass of 100 tons C ha^-1^. Their calculation reports an expected average emission of 242 million t CO_2_ year^-1^ through 2035, the end of their period under study. Over the same time frame, we find average annual emissions range from 279 to 1,000 million t CO_2_ year^-1^. Our higher estimates reflect our biomass inputs [5], the use of three scenarios, more detailed accounting of carbon losses, and increased complexity of feedbacks that affect deforestation within both human and natural systems. Note that emissions increase as deforestation increases and also the uncertainty in emissions estimates increases.

In an economic study published long before the DRC REDD+ reference levels mentioned above, Strassburg et al. [47], using data sets from the late 1990s and early 2000s, estimated that reference emission levels from DRC deforestation were 67 million t C year^-1^. Their study assumed all forested areas have an aboveground biomass of 100 tons C ha^-1^; however, we find that the biomass in areas most likely to be deforested is 77 to 82 tons C ha^-1^. Their study estimated approximately the same future emissions as the newly established reference levels of 245 million t CO_2_ year^-1^ [12]. As further comparison, the global economic analysis by Strassburg et al. [47] of the DRC’s REDD+ programs estimated that future emissions could reach 104 million t C year^-1^. Our study allows for spatial allocation of deforestation, so we simulate that deforestation will advance into forests with greater carbon density as more peripheral areas are cleared. As a result, we estimate higher emissions than Strassburg et al. [43] from our scenarios. Our calculations show a range of carbon emissions from 76 to 272 million t C year^-1^ by 2035. Comparison of Strassburg’s results with our own show agreement in the order of magnitude of emissions. Differences arise from the interactive effects of our study, which (i) uses a continuous spatial dataset of aboveground carbon biomass rather than assuming a fixed emission rate for all forested areas; (ii) accounts for savanna losses; and (iii) utilizes more recent, spatially explicit deforestation estimates.

These scenarios illustrate that conservation and development policies that mitigate pressures for new deforestation can effectively stabilize or even reduce carbon emissions. By contrast, if major efforts in economic development favor large scale agricultural expansion, such as foreign land investments or biofuel production, emissions will rise significantly. The future may hold some combination of both large scale development and sustainable conservation policies. Ideally, careful planning under a REDD+ framework should prevent the expansion of industrial agriculture into high carbon forests, and reduce future carbon emissions.

### Opportunities for Reduced Emissions

Our findings suggest that the DRC’s proposed REDD+ policies can be effective in reducing carbon emissions by increasing forest cover to offset losses elsewhere, or by replacing the need for forest clearing. Overall, the reduced rate of deforestation contributes to lower emissions through sustainable management of forests, controls on illegal logging, rehabilitation of farmlands, and increasing protected areas. Emissions are further reduced by shifting deforestation to degraded forests and woodlands that are marginal, or contain little carbon. However, it is important to investigate the impact on biodiversity, water and nutrient cycling, and ecosystem services (e.g., honey production, firewood, game) that removal of any aboveground biomass might have. Finally, a shift in population growth toward largely urban areas may reduce pressures on forests if effective policies are introduced to decrease the demand for charcoal. This will require (i) improved access to gas and electricity, and more efficient stoves; (ii) increased farming productivity, through improved access to green fertilizer, seeds, and clean water; and (iii) better education overall [12]. Currently, 95 percent of the DRC’s energy supply comes from charcoal, so reducing its use will require extensive efforts to support alternative energy sources and increase energy efficiency in urban areas [12].

## Conclusions

The DRC faces a difficult challenge to support development and improve livelihoods in a manner that ensures environmental sustainability. It is unlikely that historically low rates of deforestation can persist in the face of growing pressures to clear land due to increases in population, greater demand for wood and charcoal, cropping with reduced fallow periods leading to soil degradation, and international interests in large scale land investments for oil, biofuel and other crops. As a result, development policies should possess an understanding of impacts on forests, carbon storage and ecosystems services as they plan for sociopolitical and economic sustainability.

Passive protection will not preserve the DRC’s forests, nor promote carbon storage, nor reduce emissions. We have illustrated that the DRC’s REDD+ readiness plan, if enacted, could largely mitigate future carbon emissions. The economic pay-off for this carbon storage through international funds for climate change mitigation (e.g., the Warsaw Framework for REDD+), could provide valuable investment in the DRC, its people, and their well-being, even as it conserved much of the world’s second largest tropical forest ecosystem. Enactment of the REDD+ plan will require more analysis to target where specific conservation efforts should be focused. For example, our work shows that land near rivers is more likely to be deforested, and this could lead to water quality issues. The stakeholders involved should seek mechanisms that encourage low rates of deforestation as they locate new conservation areas and “no-deforestation” zones, and implement other guidelines. These may be informed, in part, by scenarios of carbon loss—where is the greatest amount of carbon stored and how may it best be protected? Other factors should also be considered, including biodiversity, local needs for forest products, and maintenance of forest-friendly livelihoods.

If a REDD+ policy is adopted, monitoring, although crucial, will be challenging. The region is characterized by some of the greatest cloud cover and lowest satellite coverage in the tropics. Future satellite missions should incorporate the need to provide high frequency observations if deforestation is to be monitored on an ongoing basis. Further, in-country capacity should be developed to support monitoring efforts. Increased capacity for active monitoring is already underway through a partnership between the DRC, the United Nations Food and Agriculture Organisation, and the Brazilian Space Agency (INPE), with funding from the UN-REDD program. Finally, the SimCongo model can be used as a territorial planning tool to help direct efforts to curb deforestation proactively.

## Supporting Information

S1 File All SI text and figures. (DOCX)
