# Supplementary material for: Will Passive Protection Save Congo Forests?
Source: PLoS One. 2015 Jun 24;10(6):e0128473. doi: 10.1371/journal.pone.0128473 (PMC4481311; doi:10.1371/journal.pone.0128473)
Supplement: S1 File — (DOCX) [file pone.0128473.s001.docx]

**Supplementary Material**

**Transition matrix**

We use a multi-step transition matrix derived from an Ergodic matrix that has real number Eigen values and vectors to describe a system that changes over discrete time increments (one year), where the value of any variable in a given period is the sum of fixed percentages of values of all variables in the previous time step. The transition rates set the net quantity of changes (net rates), or the percentage of land that will change to another state. In turn, gross rates are specified by unit area per unit of time (e.g., km^2^ per year). Dinamica EGO converts gross rates into net rate, dividing the extent of change by the fraction of each land use and cover class prior to change [R1]. An example of the Historical Trends transition matrix is shown in Figure A.

**Weights of evidence**

Weights of Evidence (WoE) is a Bayesian method traditionally used to derive favorability maps for spatial point phenomena [R1-R4]. In Dinamica EGO, WoE produces a transition probability map depicting the most likely areas for a change [R2, R3]. The submodels that create deforestation expansion and patches use the WoE to determine the most favorable locations for change. WoE requires categorical data, so all continuous variable maps must be categorized while preserving the data structure, as detailed by Soares-Filho et al. [R2, R3]. For each categorical range within a given variable, a coefficient and contrast, and measures of attraction or repelling effects, are calculated. SimCongo then uses these coefficients and contrasts to produce a transition probability map. This process is repeated for each land cover and land use transition, thus accounting for deforestation and cropland. An example of the WoE for the variable “distance to roads” and its correlation with deforestation is shown in Figure B.

**Selecting spatially independent input variables**

In addition, the Weights of Evidence of Correlation calculation preforms pairwise tests for categorical maps in order to test the independence assumption [R1]. Methods employed are Chi^2^, Crammers, Contingency, Entropy and Uncertainty Joint Information (Bonham-Carter, 1994). The Cramer’s coefficient and the contingency coefficient are based on Chi^2^ values and provide an exploratory and descriptive measure of spatial correlation between the maps. Both have a value of 0 for independence and a maximum value of less than 1. Crammer’s coefficient depends on Chi^2^, total area, and the area of table dimensions. The contingency coefficient depends on chi-square and total area. The entropy measure can be used to gauge associations and is a dimensionless proportion, therefore it is unaffected by measurement units. Joint information uncertainty is a measure of association between the two variables and varies between 0 (independence) and 1 (completely dependent). The joint information uncertainty is a symmetrical combination of two uncertainty measures. Although there is no agreement on what limit one should use to exclude a variable, all tests reveal low correlation for these pairs of variables. If two data sets were highly correlated in several independent tests we would have to exclude one of these. To finalize the input variables, we removed one variable from a pair if the Crammer pair was greater than 45% [R5] (Table A). We selected against the variable with high correlation to the other input variables, which was sufficient to create a set of input variables.

**Spatial simulation model**

The spatial simulation model allocates deforestation to pixels based on the spatial weight of the input variables. At the cell level, the model runs both expansion from existing deforestation and the formation of new patches of deforestation through a seeding mechanism. During patch formation, the model searches neighboring cells in order to make a joint transition. First, a core cell of the new patch is selected and then a specific number of neighboring cells are selected according to their transition probabilities. To maintain spatial integrity across biomes, the spatial variables (e.g., distance to previously cleared land) are updated annually across the entire country [R1].

**Population Density Decay Function**

We required an equation to relate population density to deforestation. To do this, we regressed observed population density by province [R6] with observed historical deforestation [R7] using the LabFit Pro software to select the model equation with the greatest fit. In this case, it is a linear function of form y = A * X + B with an R^2^ of 0.65 as the basis of Equation 4 in the main text. The model fit is shown in Figure C.

**Validation**

For validation of the model, we utilized a fuzzy similarity test to compare land uses observed in 2010 with a map simulated for 2010 derived from SimCongo’s results when initialized in 2005 [R8]. The fuzzy similarity test is based on the concept of fuzziness of location, in which a representation of a cell is influenced not only by the cell itself but, to a lesser extent, by the cells in its neighborhood (Hagen, 2003). In the fuzzy similarity test, we weighted neighboring cells using an exponential distance decay function to test spatial patterns. Lastly, we compared our results with those of a null model. This consisted of running the model with a constant transition probability map. For the HT parameterization, we found a fit of 78% within a 3.5 km search radius, which improved to a 90% fit with a 5.5 km search radius (Figure D). The search starts at the center of the cell and moves 7 pixels total, or 3.5 pixels in each direction, corresponding to a 5.5 km search radius. The HT is the only scenario that can be validated in this way: running the CON and AD scenarios from 1990 to 2010 would certainly deviate from the observed 2010 data, since these scenarios are designed to vary from historical trends.

**References**

R1. Soares-Filho, B.S., H. Rodrigues, and M. Follador, *A hybrid analytical-heuristic method for calibrating land-use change models.* Environmental Modelling & Software, 2012. In Press(Available online 16 Feb 2013).

R2. Maia, S.M.F., et al., *Effect of grassland management on soil carbon sequestration in Rondônia and Mato Grosso states, Brazil.* Geoderma, 2009. 149: p. 84-91.

R3. Galdos, M.V., et al., *Net greenhouse gas fluxes in Brazilian ethanol production systems.* GCB Bioenergy, 2010. 2(1): p. 37-44.

R4. Soares-Filho, B., et al., *Role of Brazilian Amazon protected areas in climate change mitigation.* Proceedings of the National Academy of Sciences, 2010. 107: p. 10821-26.

R5. Soares-Filho, B., H. Rodrigues, and W.L.S. Costa, *Modeling environmental dynamics with Dinamica EGO*, U.F.d.M. Gerais, Editor 2009, Centro de Sensoriamento Remoto: Belo Horizonte, Brazil.

R6. Tatem, A. and C. Linard, *Population mapping of poor countries.* Nature, 2011. 474(7349): p. 36-36.

R7. Potapov, P.V., et al., *Quantifying forest cover loss in Democratic Republic of the Congo, 2000-2010, with Landsat ETM+ data.* Remote Sensing of Environment, 2012. 122: p. 106-116.

R8. Almedia, C., et al., *Neural networks and cellular automata for modeling intra-urban land use dynamics.* International Journal of Geographical Information Science 2008. 22(9): p. 943-963.

**Captions and Figures**

**Fig. A. Historical Trends transition matrix**

| **Transition** | **Rate** |
| --- | --- |
| Forest to deforested | 0.000631 |
| Woodland to deforested | 0.001122 |

**Figure B.** **Example visualization of WoE,** showing deforestation is positively correlated to distance to roads (x-axis, meters) for areas close to roads and becomes increasingly negatively correlated as distance increases.


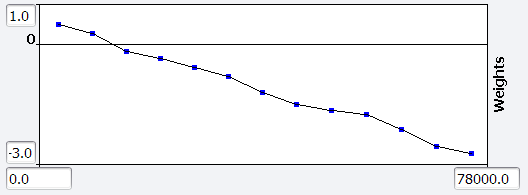


**Table A**. **Example of spatial auto-correlation analysis** for one variable, biomass, and the transition from forested to deforested areas. This analysis is repeated for each transition and each variable combination. This example shows all variables had a Crammer value of less than 45% and were acceptable for use in SimCongo.

| Transition:  Forest->Deforested | |  |  |  |  |  |
| --- | --- | --- | --- | --- | --- | --- |
|  |  | ----- Crammer ------ | | | ------ Entropy ----- | |
| First  Variable | Second  Variable | Chi^2^ | Crammer* | Contingency | Joint  Entropy | Joint Information  Uncertainty |
| ------------- | ------------------- | ----------------------------- | | | ----------------- | |
| Biomass | Croplands | 68,410 | 0.21 | 0.43 | 2.25 | 0.11 |
| Biomass | Distance to deforestation | 16,794 | 0.11 | 0.23 | 2.73 | 0.02 |
| Biomass | Population density | 6,409 | 0.05 | 0.15 | 1.66 | 0.01 |
| Biomass | Railway distance | 20,137 | 0.09 | 0.25 | 3.61 | 0.02 |
| Biomass | Rivers distance | 30,088 | 0.11 | 0.31 | 3.49 | 0.03 |
| Biomass | Roads distance | 53,584 | 0.15 | 0.39 | 4.52 | 0.04 |
| Biomass | Slope percent | 2,706 | 0.06 | 0.10 | 1.87 | 0.01 |
| Biomass | Soil | 189,758 | 0.28 | 0.62 | 3.46 | 0.13 |
| Biomass | Towns distance | 15,680 | 0.08 | 0.22 | 4.39 | 0.014 |
| Biomass | Vegetation | 267,712 | 0.34 | 0.69 | 2.98 | 0.22 |
| Biomass | Villages distance | 21,276 | 0.10 | 0.26 | 2.78 | 0.03 |

**Figure C**. **Deforestation correlation with population density** used to derive a linear relationship between the independent and dependent variable.


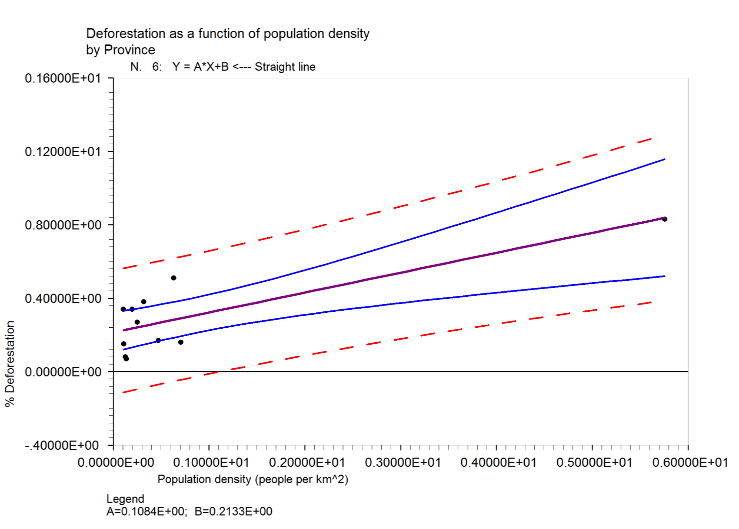


**Figure D. Fuzzy similarity results** for observed 2010 land cover and simulated 2010 land cover by SimCongo initialized at 2005. A value of 0.9 with a key of 11 represents a 90% agreement between the models at a window width of 11 pixels, or a radius of 5.5 km.

**
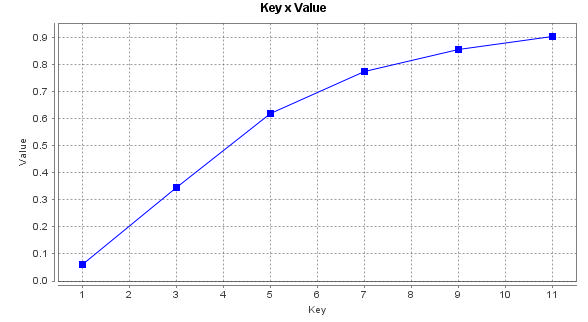
**
